# Preparation and Properties of Partial-Degradable ZrO_2_–Chitosan Particles–GelMA Composite Scaffolds

**DOI:** 10.3390/polym14194233

**Published:** 2022-10-09

**Authors:** Yang Ji, Mengdie Hou, Jin Zhang, Meiqi Jin, Tianlin Wang, Huazhe Yang, Xiaodong Zhang

**Affiliations:** 1 Department of Stomatology, General Hospital of Northern Theater Command, Shenyang 110016, China; 2 School of Intelligent Medicine, China Medical University, Shenyang 110122, China

**Keywords:** porous zirconia matrix, chitosan particles, GelMA, composite scaffold

## Abstract

In the field of bone repair, the inorganic–organic composite scaffold is a promising strategy for mimicking the compositions of the natural bone. In addition, as implants for repairing load-bearing sites, an inert permanent bone substitute composites with bioactive degradable ingredients may make full use of the composite scaffold. Herein, the porous zirconia (ZrO_2_) matrix was prepared via the template replication method, and the partial degradable ZrO_2_–chitosan particles–GelMA composite scaffolds with different chitosan/GelMA volume ratios were prepared through the vacuum infiltration method. Dynamic light scattering (DLS) and the scanning electron microscope (SEM) were adopted to observe the size of the chitosan particles and the morphologies of the composites scaffold. The mechanical properties, swelling properties, and degradation properties of the composite scaffolds were also characterized by the mechanical properties testing machine and immersion tests. The CCK-8 assay was adopted to test the biocompatibility of the composite scaffold preliminarily. The results show that chitosan particles as small as 60 nm were obtained. In addition, the ratio of chitosan/GelMA can influence the mechanical properties and the swelling and degradation behaviors of the composites scaffold. Furthermore, improved cell proliferation performance was obtained for the composite scaffolds.

## 1. Introduction

Nowadays, the large bone defect at load-bearing sites caused by trauma, tumors, etc., is still a major clinical problem, and the existing ways to deal with the problem are unsatisfactory due to various restrictions. Autologous bone grafting, a well-known “gold standard” for the treatment of bone defects, has a limited source of autologous bone and is difficult to extract to repair large bone defects [1]. Allogeneic bone grafting has a potential high infection rate and the risk of immune rejection. Therefore, the construction of suitable bone substitutes that can perform the functions of natural bone is of great importance for the repair of the bone defects [2,3,4,5]. From this perspective, mimicking the compositions (inorganic–organic compositions) and behaviors (degradation during the metabolism) is considered as one of the most promising feasible strategies. Besides, taking into account that the fully degradable implant may not be suitable for repairing the load-bearing large bone defect, and the potential bacterial infections during the bone graft surgery, the construction of a scaffold having an inert permanent bone matrix composite with ingredients that have various bioactivities (antibacterial activity, etc.) is remarkable as an “ideal” strategy. 

Zirconia (ZrO_2_), a nondegradable inert bioceramic, has excellent biocompatibility, thermal stability, corrosion-resistance, and mechanical properties, making it a desirable permanent bone scaffold for repairing load-bearing sites [6,7]. To date, various techniques have been carried out to prepare scaffolds, including electrostatic spinning [8,9], template replication [10], sol-gel [11,12], freeze-drying [13,14], pore-forming [15], and 3D printing [16,17]. As an ideal bone tissue engineering scaffold, it is important to be tunable in terms of the size and shape of the scaffold to meet the demands of bone defects sites. It is also favorable to provide a suitable pore size and pore structure to facilitate the growth of bone tissue, and to provide space for the circulation of products generated by cell growth, migration, and metabolism. Meanwhile, considering that the porosity of human cancellous bone ranges from 50% to 90%, the template replication method is preferable because it is simple to operate, and the pore size and porosity can be tunable according to different templates. However, the inertness of ZrO_2_ is unfavorable to provide a good microenvironment for activities such as cell growth and metabolism, and an ideal bone tissue engineering scaffold is considered to mimic the structure and properties of the extracellular matrix (ECM).

Hydrogel, a type of material that has similar structures and properties to ECM, is considered an excellent cell scaffold that can facilitate cell migration and growth, as well as the transport of materials within the organism [18,19,20,21]. Especially, methacrylate gelatin (GelMA) hydrogels have attracted great concern as a cell scaffold due to their excellent biocompatibility, bioactivities, and enzymatic degradation properties. It can facilitate good environment for cell adhesion, proliferation, and differentiation. However, the mechanical properties of GelMA are insufficient for repairing large bone defects at load-bearing sites. Therefore, it is feasible to composite the GelMA hydrogel with a rigid matrix such as ZrO_2_ or titanium. Besides, as a natural polysaccharide material, chitosan has good biocompatibility, biodegradability, bioadhesion, natural antimicrobial and antitumour properties, etc. It has been widely applied in drug-delivery system carriers for sustained release, controlled release, and targeted formulations of drugs [22,23,24,25]. It is known that the morphologies of chitosan can influence the release of drugs, especially chitosan particles, which may have unique functions on drug delivery and bioactivities. Furthermore, chitosan particles derived from the ionic gelation method have a reinforced polymeric network through ionic cross-linking between chitosan and sodium tripolyphosphate, which is beneficial to control the release behavior of drugs. Additionally, it is considered that the addition of chitosan can reduce the probability of infection due to its antimicrobial properties, which are also beneficial for bone graft surgery [26,27]. Accordingly, it is speculated that the addition of chitosan particles into the artificial permanent bone scaffold can further functionalize the scaffold, which may endow the scaffold with antibacterial activities and the potential function of drug-loading and controlled release. However, to the best of our knowledge, there is no research on the construction of partial-degradable ceramic-chitosan particles-hydrogel systems as multifunctional bone scaffolds.

Herein, a partial degradable ZrO_2_–chitosan particles–GelMA composite was devised and settled as a multifunctional bone scaffold. The porous zirconia is prepared as a matrix for permanent bone implants to repair the large bone defects at load-bearing sites due to its good mechanical properties, biocompatibility, and corrosion resistance. GelMA hydrogel was adopted to facilitate good environment for cell activities, and chitosan particles were embedded into the GelMA hydrogel to further functionalize the composite. The mechanical properties, swelling properties, and degradation properties of the composite scaffolds were characterized to preliminarily test the feasibility of the composite scaffold, and this work may provide a promising strategy for bone repairing at load-bearing sites.

## 2. Materials and Methods

### 2.1. Materials

The polyurethane sponges (60 ppi) templates were purchased from Nantong Yirilong Chemical Science Co., Ltd. (Nantong, China), and the sponges were cut into blocks of 1 cm × 1 cm × 0.5 cm and soaked into a 2 M sodium hydroxide solution for 8 h to remove impurities from the template. Afterwards, they were washed with deionized water ultrasonically three times, followed by air-drying. Poly (Vinyl Alcohol) was purchased from Damas-beta (Shanghai, China), and yttria-stabilized tetragonal zirconia polycrystal (Y-TZP) was purchased from Shanghai Aladdin Bio-Chem Technology Co., Ltd. (Shanghai, China). Sodium hydroxide (NaOH) was purchased from Beijing Chemical Plant (Beijing, China). Chitosan (≥75% deacetylation) and methacrylic anhydride (MA) were purchased from Sigma-Aldrich (St. Louis, MO, USA). Sodium tripolyphosphate and glacial acetic acid were purchased from Macklin Inc., China (Shanghai, China). Gelatin and photoinitiator (Irgacure 2959, 98%) were purchased from Shanghai Yuanye Biotechnology Co. (Shanghai, China). Collagenase Type II (Collagenase) was purchased from Beijing Solaibao Technology Co., Ltd. (Beijing, China), and dialysis tube (10K MWCO, 22 mm) was purchased from Thermo Fisher Scientific (Rockford, IL, USA). All of the above reagents were used as received without further purification.

### 2.2. Methods

**Figure 1:**
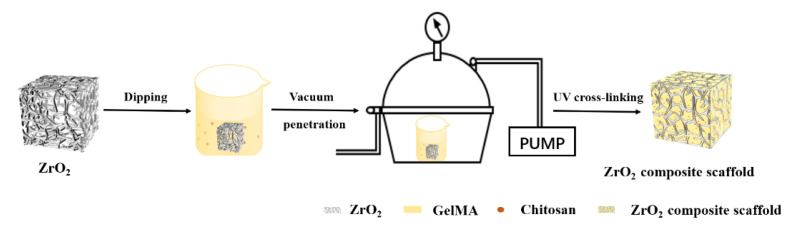
Schematic flowchart of the preparation of the ZrO2–chitosan particles–GelMA composite scaffold.

The schematic flowchart for the preparation of the composite porous ZrO_2_–chitosan particles–GelMA hydrogel is shown in Figure 1.

#### 2.2.1. Preparation of Porous Zirconia Matrixes

The slurry was obtained by mixing 70 wt% Y-TZP powder with 30 wt% deionized water and 0.1 wt% polyvinyl alcohol (PVA). The pre-treated polyurethane sponge blocks were immersed into the slurry for 5 s, and the excess slurry was taken out and removed. Afterwards, the Y-TZP containing templates were dried at 80 °C for 12 h, followed by two sintering stages: (1) increasing the sintering temperature to 800 °C at a rate of 2 °C/min, maintaining the temperature for 2 h to remove the polyurethane templates; and (2) raising the sintering temperature to 1500 °C at a rate of 3 °C/min and maintain the temperature for 3 h. Then, the white porous ZrO_2_ matrixes were obtained after cooling.

#### 2.2.2. Preparation of Chitosan Particles

In total, 50 mg of chitosan powders was mixed into 20 mL of 1% glacial acetic acid solution and stirred (500 rpm at 25 °C) continually to form a chitosan–acetic acid solution. The pH of the solution was adjusted to 5.5 with 2 M of NaOH. Meanwhile, sodium tripolyphosphate was dissolved into deionized water to make a 1 mg/mL solution, and the sodium tripolyphosphate solution with different volumes, i.e., 6 mL, 8 mL, 12 mL, 12.5 mL and 17 mL, was added dropwise to the chitosan–acetate solution using a micro syringe pump at a rate of 0.2 mL/min to obtain a solution with different mass ratios of chitosan and sodium tripolyphosphate. After adding the sodium tripolyphosphate solution, the stirring process was still maintained for another 10 min. Finally, a light blue opalescence could be observed in the solution. 

#### 2.2.3. Synthesis of GelMA Prepolymer

The synthesis method of GelMA prepolymer was described elsewhere [28], and the details was as follows: 10 g of gelatin was dissolved in 200 mL of PBS (pH = 7.4), stirring continuously (240 rpm) at 50 °C until a homogeneous solution was obtained. Afterwards, 16 mL of MA was added dropwise into the gelatin solution using a micro syringe pump at a rate of 0.2 mL/min, and the mixed solution was stirred at the same rate and temperature for 2 h. The solution was then diluted by adding 200 mL of PBS solution. After continued stirring for 10 min, the solution was placed into dialysis tubes and dialyzed in deionized water for 10 days to remove the by-products and unreacted MA from the solution. At the end of dialysis, 400 mL of deionized water was added to the solution and stirred continuously for 15 min. Subsequently, the solution was packed into 10 mL centrifuge tubes and the samples were lyophilized for 4 days to obtain a white spongy GelMA prepolymer. 

#### 2.2.4. Preparation of Porous Zirconia–Chitosan Particles–Composite Scaffolds

The chitosan–acetate solution containing chitosan particles was injected into the porous ZrO_2_ matrixes by a syringe, and three vacuum infiltration steps were performed for 15 min each to make the solution fully infiltrate and fill the ZrO_2_ matrixes. Next, 0.3 M sodium hydroxide solution was adopted to cure the solution for 30 s, and the porous zirconia–chitosan particles’ composite scaffolds were placed in a freezer at −80 °C for two days, followed by freeze-drying for two days.

#### 2.2.5. Preparation of Porous Zirconia–Chitosan Particles–GelMA Hydrogel Composite Scaffolds

Different amounts of GelMA prepolymer were dissolved in PBS containing 1% (*w/v*) photoinitiator at room temperature under ultrasonic agitation to form 5% (*w/v*), 10% (*w/v*), 15% (*w/v*), and 20% (*w/v*) GelMA prepolymer solutions, respectively. Meanwhile, chitosan particles whose ratio of chitosan/sodium tripolyphosphate solution was 50:17 (m/v) were separated from the chitosan–acetate solution in a 10 mL centrifuge tube through centrifugation, followed by adding the deionized water to 1ml for each centrifuge tube to obtain chitosan particle suspension. Afterwards, GelMA solution containing chitosan particles was prepared with the volume ratios of GelMA/chitosan of 10:3. The solutions were infiltrated and filled into the ZrO_2_ matrix by vacuum infiltration as described in Section 2.2.4. Next, GelMA was cured by UV with a UV lamp (10 mw/cm^2^) at a wavelength of 365 nm for 5 min to form the GelMA hydrogel, and the ZrO2–chitosan particles–GelMA composite scaffolds were obtained.

### 2.3. Characterizations

The structure and morphology of the scaffolds and microspheres were observed by a scanning electron microscope (SEM, JSM-7001F, JEOL, Tokyo, Japan). Dynamic light scattering (DLS) was performed with Zetasizer Nano ZS90 (Malvern, UK) to study the size of chitosan particles.

The dry weight of the ZrO_2_–chitosan particles–GelMA composite scaffolds was weighed and recorded as W_d_. The scaffolds were then immersed in PBS buffer solution and swelled for different time intervals (1, 2, 4, 8, 12, 24, and 48 h), the excess water was removed from the surface of the scaffolds with filter paper, and the mass W_s_ was recorded. The swelling ratio was calculated according to the following equation:

Swelling ratio = (W_s_ − W_d_)/W_d_ (1)

The degradation performances of the composite scaffolds were characterized as follows: samples were immersed in PBS at 37 °C until they reached swelling equilibrium and then weighed, and their initial mass was recorded as W_e_. The samples were then immersed in 20 μg/mL of type II collagenase PBS solution at 37 °C. The samples were removed at specific times each day; the surface solution was wiped off with absorbent paper; the mass of the sample was weighed with an electronic balance, recorded as W_t_; and the degradation rate of the samples was calculated by the following equation:

Degradation ratio = (W_e_ − W_t_)/W_e_ (2)

The mechanical properties of the composite scaffolds were tested with Mechanical Properties Testing Machine (M-3000, Care Measurement and Control Technology Ltd., Tianjin, China), and the process was as follows: the surface of the composite scaffold was smoothed with 2000 grit sandpaper to ensure uniform forces on the contacting surface. To simulate the environment in the human body, the scaffold was immersed in PBS solution to reach the swelling equilibrium and then tested for mechanical properties at a compression rate of 0.5 mm/min. Three parallel samples were taken for each group of tests.

The cell proliferation of the composite scaffold was tested with CCK-8 assay. The ZrO_2_ composite scaffold samples were washed thrice with the PBS and MEMα medium after alcohol immersion disinfection, respectively, and then the samples were cultured in MEMα medium, which contained 10% FBS and 1% P/S, in the CO_2_ incubator (37 °C, 5% CO_2_) for 24 h to form extract liquid. The resulting extraction was sterilized with 0.2 μm filter membranes and stored at 4 °C for further use, and 4 × 10^3^ MC3T3-E1 cells/well were added to each well of a 96-well plate for 24 h. After 24 h of incubation, the cell culture solution was replaced by the sample extracts for 1, 3, and 5 days. At each time point, 10 μL of CCK8 solution was added into each well. After further incubation for 2 h, a microplate reader (Bio-Rad, Model 680, Hercules, CA, USA) was used to measure the optical density of each well at 450 nm. 

All of the data in the results were analyzed using t-tests and one-way analysis of variance (ANOVA) with a significance level of *p* < 0.05.

## 3. Results

### 3.1. Size of Chitosan Particles

**Figure 2:**
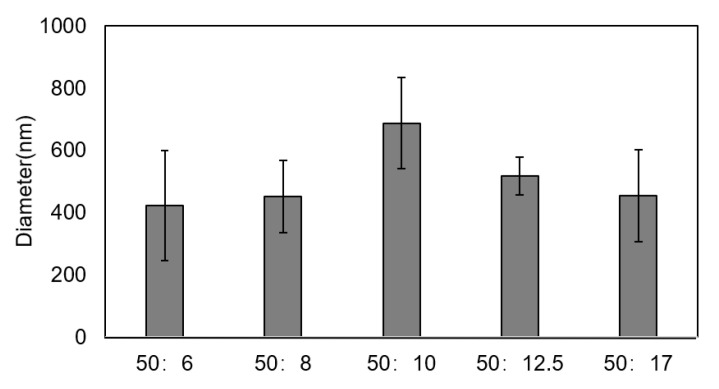
Size distributions of chitosan particles with different ratios (*w/v*) of chitosan/sodium tripolyphosphate solution.

The size distributions of the chitosan particles with a different ratio (*w/v*) of chitosan/sodium tripolyphosphate solution are shown in Figure 2, and the ratios are 50:6, 50:8, 50:10, 50:12.5, and 50:17. It can be seen that there are no obvious differences in the particle sizes for different ratios, and the particle sizes of samples for the five different ratios were all in the range of 400–700 nm. The formation of chitosan particles in this study lies in the process through the cross-linking of anions released by sodium tripolyphosphate and cations released by chitosan. Accordingly, the particle sizes of chitosan are remarkably affected by the ionic cross-linking process. When the concentration of anions is close to that of cations, excess sodium tripolyphosphate has little effect on particle size since there is no further ion cross-ling process taking place. As a result, the ratio (*w/v*) of chitosan/sodium tripolyphosphate solution had little effect on the particle sizes. During the subsequent experiment to separate the chitosan particles, it was found that although the size of the particles was small for the samples, with ratio of 50:6 and 50:8, it was difficult to re-disperse the microspheres into the solution after the centrifugation. The sample with a ratio of 50:17 had a relatively small average particle size as well as a narrow size distribution of the particle, which hints that chitosan nanoparticles have good monodispersity. Therefore, we chose the ratio of 50:17 to prepare the composite scaffold in the subsequent experiments. It is also interesting that the particle size for the sample whose ratio is 50:12.5 is a little greater than the sizes for samples with ratios of 50:6, 50:8, and 50:17 solutions, and a study on the relative reason is in progress.

The size distributions of the chitosan particles with a different ratio (*w/v*) of chitosan/sodium tripolyphosphate solution are shown in Figure 2, and the ratios are 50:6, 50:8, 50:10, 50:12.5, and 50:17. It can be seen that there are no obvious differences in the particle sizes for different ratios, and the particle sizes of samples for the five different ratios were all in the range of 400–700 nm. The formation of chitosan particles in this study lies in the process through the cross-linking of anions released by sodium tripolyphosphate and cations released by chitosan. Accordingly, the particle sizes of chitosan are remarkably affected by the ionic cross-linking process. When the concentration of anions is close to that of cations, excess sodium tripolyphosphate has little effect on particle size since there is no further ion cross-ling process taking place. As a result, the ratio (*w/v*) of chitosan/sodium tripolyphosphate solution had little effect on the particle sizes. During the subsequent experiment to separate the chitosan particles, it was found that although the size of the particles was small for the samples, with ratio of 50:6 and 50:8, it was difficult to re-disperse the microspheres into the solution after the centrifugation. The sample with a ratio of 50:17 had a relatively small average particle size as well as a narrow size distribution of the particle, which hints that chitosan nanoparticles have good monodispersity. Therefore, we chose the ratio of 50:17 to prepare the composite scaffold in the subsequent experiments. It is also interesting that the particle size for the sample whose ratio is 50:12.5 is a little greater than the sizes for samples with ratios of 50:6, 50:8, and 50:17 solutions, and a study on the relative reason is in progress.

### 3.2. Morphologies and Structures of Zirconia-Based Composite Scaffolds

#### 3.2.1. ZrO_2_ Matrix and ZrO_2_–Chitosan Composite Scaffold

**Figure 3:**
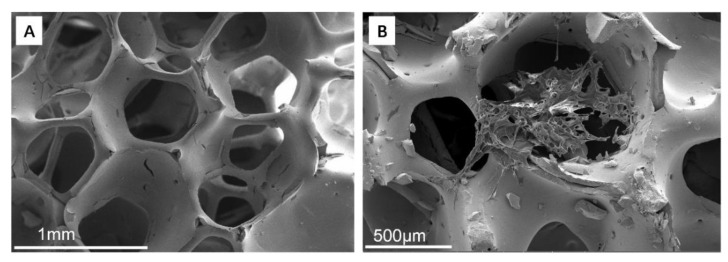
SEM images of porous zirconia matrix and ZrO_2_–chitosan composite scaffold. (**A**) porous ZrO_2_ matrix; (**B**) ZrO_2_–chitosan composite scaffold.

SEM images of porous zirconia matrix and ZrO_2_–chitosan composite scaffold are shown in Figure 3. The porous zirconia matrix had a structure of the three-dimensional interconnection network with relatively uniform pore size (Figure 3A), exhibiting an ideal structure as bone scaffolds. After infiltrating and filling the zirconia matrix with chitosan–acetate solution containing chitosan particles, it was found that the freeze-dried chitosan-based polymers exhibited a mesh-like morphology. It should be noted that the polymers had poor adhesion to the porous zirconia matrix, making the matrix partially filled with polymers. Therefore, it is necessary to composite other materials (such as GelMA hydrogel) besides chitosan to improve the adhesion between the zirconia matrix and polymers.

#### 3.2.2. ZrO_2_–GelMA Composite Scaffolds

**Figure 4:**
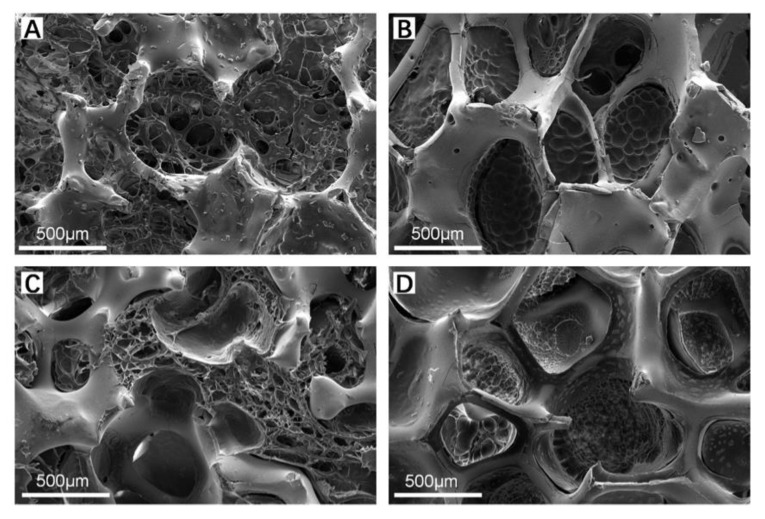
SEM images of ZrO_2_–GelMA composite scaffold. (**A**) ZrO_2_—5% GelMA; (**B**) ZrO_2_—10% GelMA; (**C**) ZrO_2_—15% GelMA; and (**D**) ZrO_2_—20% GelMA.

Figure 4A–D show SEM images of ZrO_2_–GelMA composite scaffolds filled with four different GelMA concentrations (5, 10, 15, and 20% (*w/v*), respectively). From the figure, it can be seen that the GelMA hydrogel successfully and uniformly infiltrated and filled the pores of zirconia matrixes, and the mesh-like GelMA hydrogel also had a structure of interconnection network inside the pores of zirconia matrixes. The pore size of GelMA for the composite scaffolds was (80.62 ± 17.50), (79.10 ± 16.93), (75.47 ± 24.87), and (53.70 ± 23.91) μm for hydrogel with concentrations of the 5, 10, 15, and 20% (*w/v*), respectively. It is well-known that the pore size GelMA can be affected by the degree of cross-linking of MA and gelatin. In this experiment, the cross-linking degree of MA and gelatin is same, while the increase in the concentration (viscosity) of GelMA can increase in the internal polymer chain and a higher chance of entanglement of chains [29], leading to the decrease in the pore size of hydrogel. Therefore, GelMA hydrogel was considered as a proper polymer to composite with the zirconia scaffold to obtain the ZrO_2_–GelMA composite scaffold.

#### 3.2.3. ZrO_2_–Chitosan Particles–GelMA Composite Scaffolds

**Figure 5:**
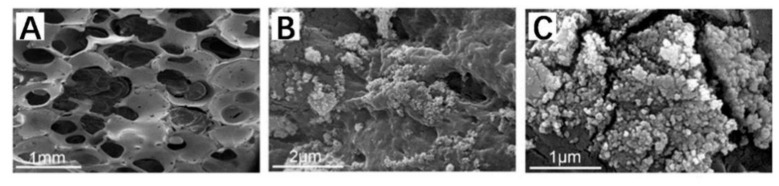
SEM images of the ZrO_2_–chitosan particles–GelMA composite scaffold. (**A**) ZrO_2_–chitosan particles–GelMA composite scaffold; (**B**) chitosan particles–GelMA inside the pores of scaffold; and (**C**) enlarged image of chitosan particles.

SEM images of the ZrO_2_–chitosan particles–GelMA (ZrO_2_–CS–GelMA) composite scaffold are shown in Figure 5. Figure 5A shows the overall morphology of the composite scaffold, and Figure 5B,C show the chitosan particles–GelMA inside the pores of the scaffold and the enlarged image of the chitosan particles, respectively. It can be seen that chitosan particles–GelMA could infiltrate and fill in the pores of the zirconia matrix successfully and that the distribution of the chitosan-containing GelMA hydrogel in the pores is uniform. Additionally, the chitosan particles can be embedded well in GelMA hydrogel, and an increasing number of chitosan particles could be found compared with the ZrO_2_–Chitosan particles composite scaffold (Figure 3B). In addition, the particle size of the chitosan was around 60 nm, as detected by Nano Measurer software, which is quite different from the values detected by DLS (around 455 nm). The difference may be due to the high water absorption capacity of the chitosan particles. In the DLS test, the chitosan particles absorbed water and swelled. In contrast, for the SEM test, the zirconia composite scaffolds were sufficiently freeze-dried to remove the water, resulting in a smaller particle size obtained in SEM images. Similar results were also found indicating that the particle size of particles measured by DLS would be relatively large.

### 3.3. Swelling Properties of Composite Scaffolds

**Figure 6:**
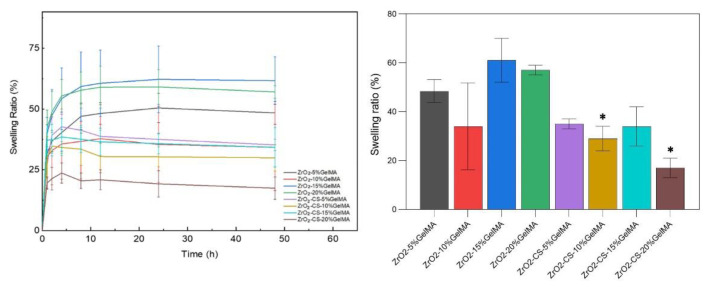
Swelling performance of zirconia-based composite scaffolds, * *p* < 0.05.

Figure 6 shows the swelling performance of ZrO_2_–GelMA and ZrO_2_–CS–GelMA composite scaffolds (the GelMA concentrations are 5% (*w/v*), 10% (*w/v*), 15% (*w/v*), and 20% (*w/v*), respectively). All of the ZrO_2_–GelMA scaffolds with different concentrations can swell rapidly within 8 h of immersion in PBS solution, and it reached swelling equilibrium at 12h of immersion. The ZrO_2_–GelMA scaffold with a GelMA hydrogel concentration of 15% had the largest swelling rate, i.e., 61.68% ± 9.76%, while the scaffold whose GelMA hydrogel concentration was 10% had the lowest swelling rate (34.24% ± 17.78%). For the ZrO_2_–CS–GelMA composite scaffolds, the composite scaffolds swelled rapidly within the first 4h of immersion, reaching the swelling equilibrium at 12h of immersion. Additionally, scaffolds with GelMA concentrations of 5% and 15% had a better swelling performance, whose swelling ratio was 35.15% ± 1.97% and 34.09% ± 8.06%, respectively.

### 3.4. Degradation Properties of Zirconia Composite Scaffolds

**Figure 7:**
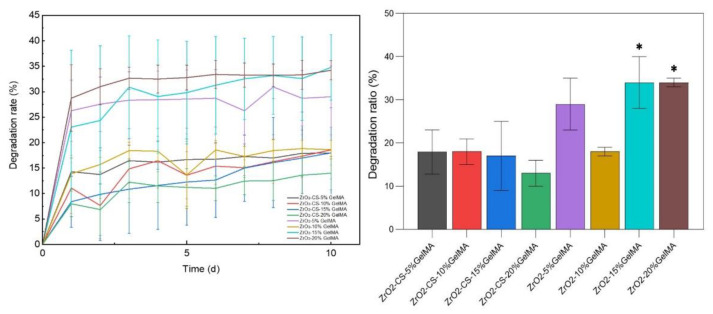
Degradation properties of zirconia composite scaffolds, * *p* < 0.05.

From Figure 7, the degradation rate of all the composite scaffolds increased noticeably in 1 d, and the overall degradation rates increased with the increase in time. For the ZrO_2_–GelMA composite scaffolds, the degradation rate of ZrO_2_–10% GelMA was relatively low (18.62% ± 1.84%), while the degradation rates for the scaffold are 29.02% ± 6.01%, 34.78% ± 6.41%, and 34.21% ± 1.86%, respectively, for the concentration of GelMA of 5%, 15% and 20%. For the ZrO_2_–CS–GelMA composite scaffolds, the degradation rates were 17.93% ± 5.11%, 18.57% ± 2.98%, 17.98% ± 8.83%, and 13.99% ± 3.32%, respectively, for the concentrations of GelMA of 5%, 15%, and 20%. Overall, the degradation rates of the composite scaffolds were significantly lower than those of the ZrO_2_–GelMA composite scaffolds. The average degradation rate of the ZrO_2_–CS–GelMA composite scaffolds was 58.72% that of the ZrO_2_–GelMA composite scaffolds. The reason for this is that the GelMA hydrogels tend to degrade quickly with the aid of collagenase, while the addition of chitosan particles can weaken the effect of collagenase on the composite polymers, which is consistent with the results of previous studies [30].

### 3.5. Mechanical Properties of Zirconia Composite Scaffolds

**Figure 8:**
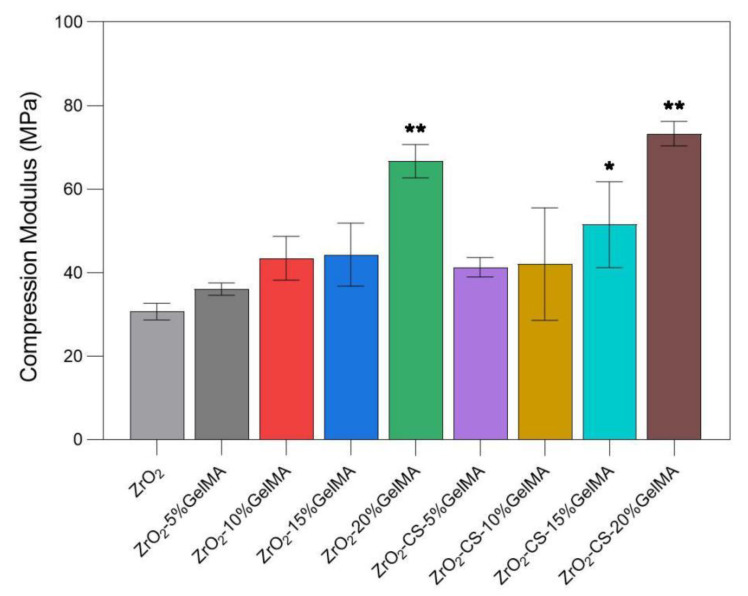
Compressive strength of porous zirconia scaffolds with composite scaffolds, * *p* < 0.05, ** *p* < 0.01, compared to ZrO_2_ group.

Shown in Figure 8, the compressive modulus of all of the composite scaffolds is greater than the neat porous ZrO_2_ scaffold, demonstrating that the composite ZrO_2_ with polymers is a feasible way to improve the mechanical properties of the scaffolds. In addition, for the ZrO_2_–GelMA scaffolds, the compressive strength of the scaffold increased gradually, and the compressive strength of the composite scaffold with a hydrogel concentration of 20% (*w/v*) was nearly twice that of the composite scaffold with a hydrogel concentration of 5% (*w/v*), increasing from (36.061 ± 1.972) MPa to (66.680 ± 4.023) MPa. Moreover, compared with ZrO_2_–GelMA, the compressive strength of the ZrO_2_–CS–GelMA composite scaffolds improved; the compressive strength was (41.312 ± 2.303) MPa, (42.047 ± 13.476) MPa, (51.503 ± 10.283) MPa, and (73.272 ± 2.949) MPa, respectively, for the concentrations of GelMA of 5%, 10%, 15%, and 20%. The results showed that the addition of chitosan particles can improve the mechanical properties of the scaffold. 

### 3.6. Cell Proliferation of Zirconia Composite Scaffolds

**Figure 9:**
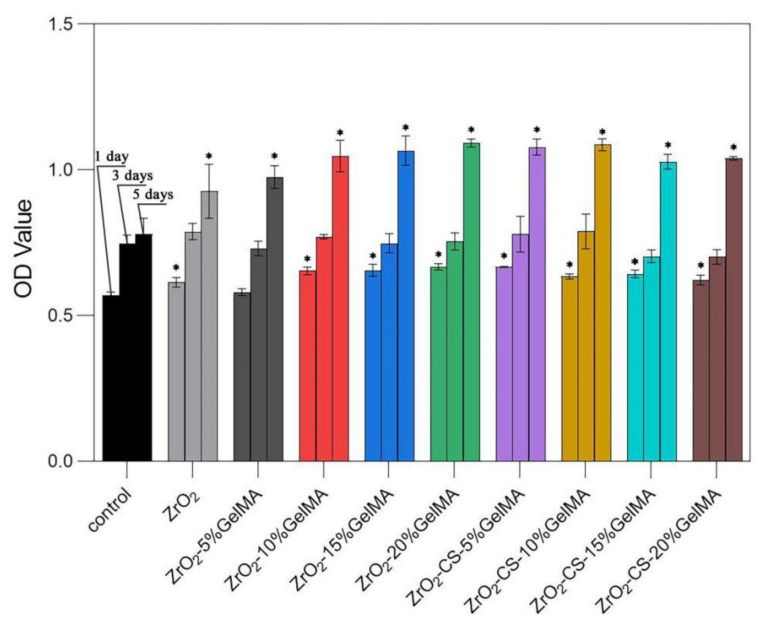
Proliferation performances of porous zirconia scaffolds with composite scaffolds, * *p* < 0.05, compared to control group.

Figure 9 shows the proliferation performances of the ZrO_2_–GelMA and ZrO_2_–CS–GelMA composite scaffolds. The number of MC3T3-E1 on both the ZrO_2_–GelMA and ZrO_2_–CS–GelMA composite scaffolds increased progressively with time. Notably, the cell proliferation of the composite scaffolds was significantly higher than that of the ZrO_2_ scaffold. Moreover, compared with ZrO_2_–GelMA, the cell proliferation of the ZrO_2_–CS–GelMA composite scaffolds was more obvious, probably due to the synergistic effects of GelMA hydrogel and chitosan particles in terms of biological activity. These results indicated that both the ZrO_2_–GelMA and ZrO_2_–CS–GelMA composite scaffolds possessed excellent proliferation of osteoblasts. In particular, the ZrO_2_–CS–GelMA composite scaffolds with 10% GelMA displayed the most positive effect on MC3T3-E1 cell proliferation.

## 4. Discussion

**Figure 10:**
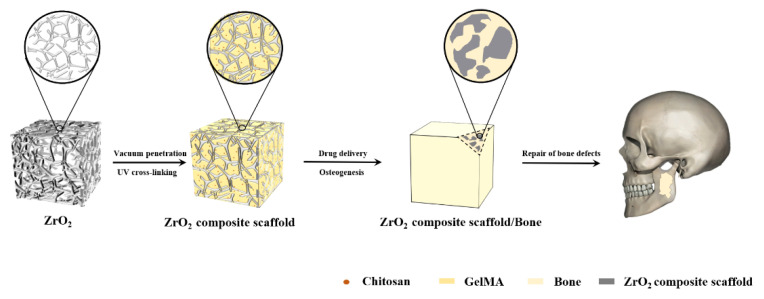
Schematic functions of the ZrO_2_–chitosan particles–GelMA composite scaffold.

In this study, we intended to devise a multifunctional bone scaffold for repairing the large bone defect at load-bearing sites. In this case, the fully-degradable scaffold cannot provide stable mechanical support and perform biological functions to repair the large bone defects. Herein, a novel scaffold named the ZrO2–chitosan particles–GelMA composite was devised and fabricated, which combines the advantages of ZrO_2_ (good mechanical properties and biocompatibilities), chitosan (good bioactivities, biodegradability and antimicrobial activity), and GelMA (excellent biological performance). Figure 10 shows the potential functions of the composite scaffold. The infiltration of chitosan particles–GelMA hydrogel can further improve the mechanical properties of the porous ZrO_2_ matrix, which can provide increased mechanical support to repair the large bone defect at load-bearing sites. Furthermore, the GelMA hydrogel can enhance the adhesion of the cells on the scaffolds and create a good environment for cell activities. Additionally, chitosan particles not only have antibacterial abilities to decrease the risk of bacterial infections but also endow the scaffold with tunable functions of drug-loading and sustaining release. As a result, the composite may be a promising multifunctional scaffold for bone repair.

In the experiment to prepare porous zirconia matrixes, 0.1 wt% PVA was added into the slurry of 70 wt% Y-TZP powder and 30 wt% deionized water. PVA is a viscous agent, which can improve the viscosity of the slurry. As a result, the slurry can be firmly stuck to the polyurethane sponge blocks, maintaining the integrity of the zirconia matrixes. 

According to the as-obtained results, it is interesting that the swelling ratio of the composites scaffold decreased after mixing with chitosan, and all the chitosan-containing scaffolds had a lower swelling ratio than the ones without chitosan. The chitosan-containing scaffolds had a lower swelling ratio than the ones without chitosan. A similar phenomenon was also observed for the chitosan–GelMA IPN composite [31]. Therefore, it is speculated that a compact network can be formed for chitosan particles–GelMA hydrogel in our scaffold, leading to the decrease in the permeation and absorbance of water. It should be noted that the network of chitosan particles–GelMA may be further modified due to the confinement effect of porous zirconia matrix. Additionally, as shown in Figure 6 and Figure 7, the samples with the lowest swelling ratio have the lowest degradation rate (ZrO_2_–10% GelMA and ZrO_2_-CS-20% GelMA), and there is still no quantitative correlation between the swelling ratio and the degradation rate, to the best of our knowledge. The speculated reason is that GelMA with low concentration has a lower degree of cross-linking, which is fragile and easily degraded. Meanwhile, the sparse network brought by the lower degree of cross-linking leaves more space for the permeation and absorbance of water, leading to a higher swelling performance. Accordingly, more systematical studies should be carried out on the interaction of ZrO_2_-CS-20% GelMA on the properties (swelling ratios, degradation rate, etc.) of the composites to obtain the composites with a tunable performance. Furthermore, it is also important to reveal the relative mechanism for the interaction of the composite scaffolds.

## 5. Conclusions

ZrO_2_–chitosan particles–GelMA composite scaffolds were devised and prepared via a vacuum infiltration method. The morphologies, structures, swelling properties, degradation properties, mechanical properties, and cell proliferation performance of the composite scaffolds have been investigated. Chitosan particles–GelMA infiltrated and filled in the pores of zirconia matrix successfully, and the distribution of the chitosan-containing GelMA hydrogel in the pores was uniform. The particle size of the lyophilized chitosan was around 60 nm, compared to that for the swelling one at approximately 455 nm. In addition, for the ZrO2–GelMA scaffold, the GelMA hydrogel concentration of 15% had the largest swelling rate, i.e., 61.68% ± 9.76%, while for the ZrO_2_–Chitosan particles–GelMA composite scaffolds, the GelMA concentrations of 5% had the largest swelling ratio (35.15% ± 1.97%). The average degradation rate of the ZrO_2_–Chitosan particles–GelMA composite scaffolds was nearly half of that of the ZrO_2_–GelMA composite scaffolds. Furthermore, the introduction of hydrogels could effectively enhance the compressive strength and cell proliferation performance of the ZrO_2_ scaffolds. More systematic experiments are encouraged to investigate the biological performance of the composite scaffold.

## Data Availability

Not applicable.
